# Fructans and Fructooligosaccharides in Plants and Fruits: Metabolism, Functional Roles, and Emerging Biotechnological Opportunities

**DOI:** 10.3390/molecules31152656

**Published:** 2026-07-30

**Authors:** Luis Morales-Quintana, Patricio Ramos, Carolina Parra-Palma

**Affiliations:** 1 Multidisciplinary Agroindustry Research Laboratory, Instituto de Ciencias Biomédicas, Facultad de Ciencias de la Salud, Universidad Autónoma de Chile, Cinco Poniente #1670, Talca 3467980, Región Del Maule, Chile; luis.morales@uautonoma.cl; 2 Plant Microorganism Interaction Laboratory, Instituto de Ciencias Biológicas, Universidad de Talca, Talca 3480000, Región Del Maule, Chile; pramos@utalca.cl

**Keywords:** fruit quality, functional food, postharvest traits

## Abstract

Fructans and fructooligosaccharides (FOSs) are structurally diverse fructose-based carbohydrates synthesized from sucrose through the coordinated action of fructosyltransferases. These compounds are widely distributed in plants and have attracted increasing attention due to their dual relevance in plant physiology and human nutrition. In plants, fructans function as dynamic carbon reserves and play key roles in tolerance to abiotic stresses such as drought, cold, and salinity. In parallel, their selective fermentability and prebiotic properties have positioned them as valuable functional ingredients in food and nutraceutical applications. This review provides an updated overview of fructan and FOS metabolism, focusing on their biosynthetic pathways, structural diversity, and physiological roles in plants. Particular attention is given to the occurrence and potential functions of fructans in fruits, a topic that remains comparatively underexplored. We also discuss recent advances in strategies aimed at enhancing fructan accumulation through breeding, metabolic engineering, and synthetic biology approaches. Finally, emerging opportunities for the biotechnological exploitation of fructan metabolism are highlighted, including crop biofortification, development of functional foods, and the improvement of plant resilience to environmental stress.

## 1. Introduction

Fructans and fructooligosaccharides (FOS) are multifunctional carbohydrates that bridge plant physiology and human nutrition. In plants, they serve as dynamic carbohydrate reserves while contributing to osmoregulation, membrane stabilization, and signaling during abiotic stress [1,2,3]. Structurally, fructans exhibit considerable diversity—ranging from linear inulin-type (β 2 → 1) to branched levan-type (β 2 → 6) and mixed-type configurations—depending on plant species and enzymatic biosynthetic pathways [4,5]. This structural variability determines both physiological functions and technological applications. In temperate and drought-adapted species, fructan accumulation correlates with enhanced tolerance to cold, desiccation, and oxidative stress, while small fructan molecules may function as systemic signaling intermediates coordinating stress responses [6,7].

From a nutritional perspective, fructans and FOS are well-established prebiotics that selectively stimulate beneficial gut bacteria, including *Bifidobacterium* and *Lactobacillus*, improving digestive health, mineral absorption, and glycemic control [8,9,10]. Their biological activity is strongly structure-dependent: short-chain FOS (degree of polymerization [DP] < 10) are rapidly fermented and highly bifidogenic, whereas long-chain inulins (DP > 20) confer sustained immunomodulatory, antioxidant, and metabolic benefits [11,12,13].

Despite extensive advances in the characterization of fructan diversity and physiological roles, a critical conceptual gap persists: the absence of a coherent framework explicitly linking molecular structure → physiological function → genetic design strategy. Comparative analyses across cereals, grasses, and dicots reveal clear organ- and species-specific fructan patterns, yet the mechanistic basis connecting polymer size, branching architecture, and stress resilience remains poorly defined [14,15]. This limitation constrains rational improvement strategies aimed at integrating abiotic stress tolerance and nutritional enhancement within the same genotype—a key challenge for next-generation crop design.

Extending this conceptual gap to crop systems reveals a notable bias in current research. Most studies to date have focused on fructan accumulation in vegetative tissues—roots, stems, leaves, or tubers—where they function as storage and stress-response metabolites [7]. By contrast, fruits remain largely overlooked, despite being metabolically dynamic sink organs exposed to multiple stresses, including drought during development and oxidative damage during ripening and storage [16,17]. Their direct consumption by humans makes them ideal systems for prebiotic biofortification, enabling nutritional benefits without additional processing. Furthermore, fruit-specific promoters offer opportunities for tissue-targeted fructan engineering, minimizing potential pleiotropic effects on vegetative growth [18,19].

Recent reports confirm natural FOS presence in banana (*Musa* spp.) and strawberry (*Fragaria* × *ananassa*), validating the feasibility of fructan accumulation in reproductive tissues [20]. However, systematic understanding of (a) the genetic determinants of FOS variability among cultivars, (b) ontogenic shifts in DP profiles during ripening, and (c) the physiological role of fruit fructans in stress mitigation and postharvest longevity remains scarce.

In this context, this review addresses the need for an explicit structure–function–design framework that bridges molecular biology, physiology, and biotechnology. We propose an integrative perspective encompassing three interrelated dimensions:

1. Structure–function relationships: Defining how fructan architecture (DP, linkage type, branching) modulates plant stress physiology and prebiotic efficacy.
2. Fruit biology as a model system: Leveraging reproductive tissues to explore fructan metabolism under combined developmental and environmental cues.
3. Biotechnological translation: Connecting fundamental insights to genetic engineering and breeding strategies for crops that unite climate resilience with human health value.

By synthesizing advances in plant physiology, nutrition science, and molecular breeding, this review aims to contribute to the strategic development of climate-resilient crops with enhanced prebiotic value, reinforcing the convergence between sustainable agriculture and public health objectives.

## 2. Structural Diversity of Fructans and FOS: From Molecular Architecture to Functional Specialization

### 2.1. Structure–Function–Lineage Relationships: A Unified Framework

The structural diversity of fructans—primarily determined by glycosidic linkage pattern (β 2 → 1, β 2 → 6, or mixed) and DP—represents both evolutionary divergence and functional specialization. These structural features influence polymer solubility, molecular flexibility, hydration capacity, membrane interactions, and antioxidant properties, which in turn modulate the physiological roles of fructans under environmental stresses. Recent advances in genomics and enzymology showed that fructan composition is not randomly distributed across the plant kingdom but has evolved independently in several angiosperm lineages through diversification of GH32 fructosyltransferases [21,22,23].

Although fructan metabolism has traditionally been investigated in vegetative organs such as roots, stems, bulbs, and leaves, increasing evidence demonstrates that fructans and FOS are also present in edible fruits—including banana, strawberry, citrus, and other fleshy fruits—where they may contribute to carbohydrate partitioning, fruit development, postharvest physiology, and nutritional quality. However, compared with classical fructan-accumulating species, the functional significance of fruit-associated fructans remains largely unexplored. The following sections therefore summarize the major fructan classes according to their structural characteristics while providing representative examples across both vegetative and reproductive organs.

#### 2.1.1. Inulin-Type Fructans (Β 2 → 1 Linkage): Predominant Storage and Drought-Responsive Polymers

Inulin-type fructans consist of linear β (2 → 1)-linked fructose chains and represent the most predominant fructan class in dicotyledonous plants. They are particularly abundant in members of the *Asteraceae* (e.g., *Cichorium intybus*, *Helianthus tuberosus*, *Smallanthus sonchifolius*), but also in *Campanulaceae*, *Boraginaceae* and several *Asparagaceae* species. Beyond these classical storage organs, short-chain inulin-type fructooligosaccharides have also been identified in fruits such as banana (*Musa* spp.), strawberry (*Fragaria* × *ananassa*), and several citrus species, although their physiological roles during fruit development remain poorly understood.

The β 2 → 1 linkage provides high molecular flexibility and hydration capacity, enabling efficient osmotic adjustment during water deficit. Accordingly, numerous physiological studies have established inulin-type fructans as major contributors to drought adaptation [22]. Nemati et al. (2018) [24] demonstrated that drought-tolerant wheat cultivars accumulated higher-DP inulin-type fructans (mean DP ≈ 10), resulting in greater hydroxyl radical scavenging activity and improved membrane protection. Likewise, *Aloe vera* responds to progressive water deficit by increasing both fructan content and polymer complexity, suggesting adaptive remodeling of fructan architecture under dehydration [25].

Biosynthesis is primarily controlled by the coordinated activities of two vacuolar enzymes: sucrose:sucrose 1-fructosyltransferase (1-SST) and fructan:fructan 1-fructosyltransferase (*1-FFT*), which initiate and elongate β (2 → 1)-linked fructans within the vacuole. In *Cichorium intybus* taproots, both genes (*1-SST* and *1-FFT*) are transcriptionally induced by ABA, cold, and nitrogen limitation, leading to rapid fructan accumulation [26].

Comparative genomic analyses further revealed that amino acid substitutions within substrate-binding pockets of 1-SST and 1-FFT contribute to natural variation in polymer DP among species and genotypes [27]. Longer-DP fructans (DP 10–60) consistently showing greater osmotic adjustment and ROS scavenging compared to short-chain FOS (DP 3–10), supporting the concept that polymer length strongly influences biological function [24].

From a biotechnological perspective, the causal role of inulin-type fructans has been validated in heterologous expression studies. Co-expression of *1-SST* and *1-FFT* in non-fructan-producing species, such as potato and sugarcane, promoted inulin accumulation and enhanced drought tolerance [28,29]. These results underscore the potential of inulin-type fructans as engineering targets for developing drought-resilient, carbon-efficient crops.

#### 2.1.2. Levan-Type Fructans (Β 2 → 6 Linkage): Adaptative Polymers for Arid Environments

Levan-type fructans are characterized predominantly by β (2 → 6)-linkage fructose residues and occur mainly in monocot species adapted to arid and high-temperature environments, including members of the *Agavaceae*, *Asparagaceae*, and certain *Poaceae*. Representative species include *Agave* spp., Asparagus officinalis, and several grasses. Although true levan-type fructans have rarely been reported in fleshy fruits, fructooligosaccharides containing β (2 → 6) linkages, including neokestose and 6-kestose, have been detected in banana and citrus fruits, suggesting that elements of β (2 → 6)-based fructan metabolism also occur in reproductive tissues.

*Agave* species, which accumulate complex β 2 → 6-rich fructans (agavins) in succulent stem tissues, represent the best example of levan-type fructan specialization. These plants accumulate highly branched agavins with elevated molecular weight, allowing efficient water retention, osmotic buffering, and carbon storage under prolonged drought and extreme temperatures [30]. Although direct experimental evidence linking levan fructans to thermotolerance remains comparatively limited, their physicochemical properties suggest an important role in preserving protein function and membrane integrity under heat stress.

Levan biosynthesis requires 6-SFT (sucrose:fructan 6-fructosyltransferase) and 6G-FFT (fructan:fructan 6G-fructosyltransferase) activities (β 2 → 6 linkage formation and chain elongation, respectively). Functional studies using heterologous expression of a *6-SFT* gene from *Leymus mollis* in *Arabidopsis* demonstrated that 6-SFT-driven β 2 → 6 fructan accumulation enhanced germination and root growth under salt stress, suggesting that 6-SFT products contribute to general stress resilience pathways potentially including thermal tolerance [17].

Moreover, levan-type fructans serve multiple adaptive functions in monocots. Recent syntheses emphasize that agavins function as carbon reserves, osmotic buffers, and potentially as phloem-mobile signals coordinating whole-plant stress responses [12,30]. The highly branched structure of agavins provides adaptive versatility, combining water-binding capacity with structural stability under thermal stress.

Engineering levan biosynthesis in crops remains challenging due to the complexity of 6-SFT and 6G-FFT enzyme families and limited understanding of regulatory mechanisms. However, the demonstration that heterologous 6-SFT expression can enhance stress tolerance in model systems [17] suggests feasibility for future crop engineering targeting combined heat and drought resilience, particularly relevant for climate change adaptation.

#### 2.1.3. Graminan and Mixed-Type Fructans: Multifunctional Carbon Buffers in Cereals

Graminan-type fructans exhibit mixed β 2 → 1 and β 2 → 6 linkages, forming branched structures that combine the flexibility of inulin with the stability of levan. Predominant in temperate cereals such as wheat (*Triticum aestivum*), barley (*Hordeum vulgare*), and rye (*Secale cereale*), these polymers function as multifunctional carbon buffers supporting both stress tolerance and grain filling [23].

Unlike inulin-type fructans, graminans primarily support whole-plant carbon economy. Coordinated expression of 1-SST and 6-SFT promotes fructan accumulation in stems during vegetative growth, followed by rapid remobilization during grain filling or drought-induced carbon limitation [31]. This dynamic buffering capacity maintains reproductive development when photosynthetic carbon assimilation declines.

Functional validation has been obtained through transgenic approaches. Wheat expressing a *Ta1SST:Ta6SFT* fusion construct accumulated larger quantities of hih-DP fructans and exhibited enhanced water deficit tolerance and maintaining yield stability [32].

Metabolomic analyses have revealed tissue-specific fructan distribution within cereal grains, with short-chain fructans enriched in the aleurone and endosperm and longer polymers preferentially accumulating in embryos, indicating specialized functions in osmotic regulation and oxidative stress protection [33].

Genomic-wide association studies have further identified *1-SST*, *6-SFT*, and *1-FFT* loci linked to fructan composition variability, offering molecular markers for breeding programs targeting improved stress resilience and nutritional fiber content [23]. These discoveries provide valuable molecular markers for breeding programs seeking to improve both abiotic stress resilience and dietary fiber content in cereal crops.

#### 2.1.4. Toward a Unified Structure–Function–Lineage Matrix

**Figure 1:**
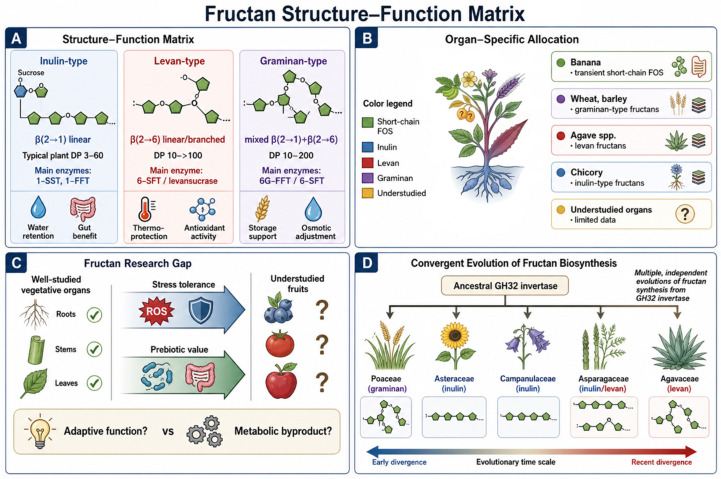
Structural diversity, biological functions, organ distribution, and evolutionary origins of plant fructans. (**A**) Structure–function matrix summarizing the main fructan classes found in plants, including inulin-, levan-, and graminan-type fructans. Differences in glycosidic linkages, degree of polymerization (DP), and key biosynthetic enzymes are associated with distinct physiological and nutritional functions, such as water retention, osmotic adjustment, antioxidant activity, storage support, and prebiotic benefits. Proposed roles in thermoprotection, particularly for levan-type fructans, are included as hypotheses requiring further experimental validation. (**B**) Representative distribution of fructan types across plant organs and species, highlighting the occurrence of short-chain fructooligosaccharides (FOS) in fruits and the predominance of specific fructan classes in crops such as wheat, barley, agave, and chicory. (**C**) Conceptual overview of current research gaps, emphasizing the extensive characterization of fructans in vegetative organs compared with the limited knowledge available for fruits, despite their potential roles in stress tolerance and human health. (**D**) Conceptual evolutionary model illustrating the proposed independent emergence of fructan biosynthetic pathways from ancestral GH32 invertases across multiple plant lineages, resulting in the diversification of fructan structures observed in modern taxa. This evolutionary framework is based on current comparative and phylogenetic evidence but remains a working hypothesis that is not yet fully validated experimentally. Created based on information synthesized from the literature reviewed in this study.

Integrating biochemical, physiological, and phylogenetic evidence yields a unified framework in which fructan architecture predicts both ecological function and biotechnological application (Figure 1A; Table 1). Inulin-type fructans are predominantly associated with osmotic adjustment and antioxidant protection in dicotyledonous storage organs but also occur as short-chain FOS in several fruits. Levan-type fructans are characteristic of monocot species adapted to arid environments and appear optimized for long-term carbon storage and environmental resilience, while β (2 → 6)-containing FOS have also been detected in some fruits. Graminan-type fructans represent an evolutionary innovation of temperate cereals, integrating multiple linkage types to maximize carbon remobilization and stress buffering.

Despite these advances, fruit fructans remain substantially less investigated than those of classical storage organs. Most available studies are limited to compositional analyses in banana, strawberry, citrus, and a few additional species, whereas mechanistic evidence linking fructan metabolism with fruit development, ripening, postharvest physiology, or fruit quality is still scarce. Expanding research toward fruit-bearing species will therefore be essential for understanding the broader biological significance of fructans and for exploiting their potential in horticultural biotechnology, functional foods, and nutritional improvement.

### 2.2. Fructan Accumulation in Fruits: An Underexplored Frontier with Dual Significance

Despite extensive research on fructan accumulation in vegetative organs (roots, tubers, stems, leaves) and cereal grains, fructan metabolism in fleshy fruits remains severely understudied (Figure 1B). This knowledge gap is particularly striking given that fruits represent a critical interface between plant stress physiology and human nutrition: fruits are reproductive organs subject to intense developmental and environmental stresses and simultaneously serve as primary dietary sources of prebiotic compounds for human health (Figure 1C). Recent evidence from limited fruit systems suggests that fructan accumulation in fruits may fulfill distinct physiological roles compared to vegetative organs, with implications for both crop improvement and nutritional enhancement (Figure 1B,C).

#### 2.2.1. Current Evidence: Which Fruits Accumulate Fructans and FOS?

Quantitative documentation of fructan and FOS accumulation in fleshy fruits remains limited and fragmented (Figure 1B,C). In contrast to vegetative storage organs such as roots, bulbs, stems, and cereal grains, only a limited number of fleshy fruit species have been investigated for endogenous fructan metabolism. Consequently, the examples presented below do not represent the diversity of fleshy fruits but rather summarize the currently available experimental evidence. This limited taxonomic coverage reflects a major knowledge gap in the literature rather than conclusive evidence that fructans are absent from most fruits. Comprehensive metabolomic, transcriptomic, and enzymatic studies across a wider range of fruit species are still needed to determine the true distribution and biological significance of fructans in fleshy fruits.

*Banana (Musa* spp.*): The best-characterized fructan-accumulating fleshy fruit*

Among fleshy fruits, banana represents the strongest and most thoroughly documented example of endogenous FOS biosynthesis. Early chromatographic analyses identified short-chain β (2 → 1)-linked FOS, including 1-kestose, nystose, and 1F-fructofuranosylnystose during ripening [38,39]. These compounds accumulate transiently as starch reserves are degraded and sucrose concentrations increase, reaching their highest levels during the mid-climacteric stages before being hydrolyzed by vacuolar fructan exohydrolases.

Molecular data indicate that this transient FOS synthesis results from partial activation of the fructan pathway. Specifically, *1-SST* expression is upregulated during early ripening, while *1-FFT* expression remains weak or undetectable, restricting fructan biosynthesis to short-chain FOS instead of long-chain inulin-type polymers [39,40]. Although these transient FOS may contribute to osmotic adjustment, membrane stabilization, or redox buffering during ripening-associated physiological changes, their precise adaptive function remains uncertain.

*Strawberry (Fragaria* × *ananassa) and tomato (Solanum lycopersicum): limited evidence for endogenous metabolism*

Beyond banana, only a few fleshy fruit species have been examined in sufficient biochemical or molecular detail. Among these, strawberry and tomato contain only trace amounts of short-chain FOS (typically of DP 3–5) during fruit development or early ripening [40]. Initial chromatographic analyses suggested the presence of compounds as 1-kestose and nystose. However, subsequent transcriptomic studies failed to detect expression of *1-SST*, *1-FFT*, or *6-SFT* orthologs in these fruits [41].

This evidence suggests that the detected FOSs are more likely generated through non-specific fructosyl transfer reactions, possibly mediated by invertases under conditions of elevated sucrose availability, rather than through a dedicated fructan biosynthetic pathway. Therefore, FOS accumulation in these species likely represents a metabolic byproduct rather than an adaptive or regulated process.

*Apple (Malus domestica) and pear (Pyrus communis): Historical reports lacking molecular support*

Early chromatographic studies from the 1980s and early 1990s detected minute levels of FOS (mainly kestose and nystose) in apple and pear tissues [42]. However, later analyses using high-resolution LC-MS and HPAEC-PAD methods did not confirm these findings, and no functional *fructosyltransferase* genes have been identified in their genomes. The earlier reports likely reflected analytical interference with sucrose and raffinose-family oligosaccharides, rather than *bona fide* fructan presence [41]. Consequently, the earlier reports are now generally considered to reflect analytical interference with sucrose or raffinose-family oligosaccharides rather than authentic fructan accumulation. Current evidence therefore indicates that fleshy fruits within the Rosaceae do not possess an active fructan metabolic pathway.

*Other reproductive organs with active fructan metabolism*

Although true fleshy fruits remain poorly represented, fructan accumulation has been extensively documented in several reproductive or edible organs that are frequently consumed but are not botanically classified as fleshy fruits.

*Asparagus* officinalis accumulates substantial amounts of inulin-type fructans in developing spears, with degrees of polymerization ranging from approximately 3 to 30 and total fructan contents reaching 10–15% of dry weight. Transcriptomic analyses demonstrate coordinated expression of 1-SST and 1-FFT during spear elongation, indicating active developmental regulation of fructan biosynthesis [26].

Similarly, *Agave* species accumulate highly branched levan-type fructans (*agavins*) in inflorescence stems and developing reproductive tissues. *Vacuolar fructosyltransferase* activities identified in *Agave tequilana* generate structurally diverse fructans with tissue- and developmental stage-specific profiles, suggesting important roles in reproductive development and adaptation to arid environments [34].

In cereal grains, which are botanically classified as caryopsis fruits, oligofructans with DP values ranging from approximately 3 to 15 accumulate in the aleurone, endosperm, and embryo. Tissue-specific fructan profiles and developmental dynamics have been described in both wheat and barley, indicating coordinated regulation of fructan metabolism during grain maturation [23,33]. Although these structures are technically fruits, their dry morphology and storage physiology differ fundamentally from those of fleshy fruits.

Overall, the available evidence is derived from a very limited number of fleshy fruit species and should therefore not be interpreted as representative of fruit diversity. Among the species investigated to date, convincing evidence for endogenous fructan biosynthesis exists only for banana, whereas other fleshy fruits generally contain low concentrations of short-chain FOS that are likely generated through non-specific metabolic reactions rather than dedicated fructan biosynthesis. In contrast, reproductive and storage organs such as asparagus spears, agave inflorescences, and cereal grains consistently exhibit active fructan metabolism. This uneven distribution suggests that fructan biosynthesis has followed distinct evolutionary trajectories among reproductive organs, with most fleshy fruits apparently relying on sucrose- and hexose-based carbohydrate allocation strategies. Future comparative metabolomic, transcriptomic, and functional studies encompassing a broader diversity of fruit species will be essential to determine whether low-level fructan accumulation in fleshy fruits represents an adaptive trait, an evolutionary remnant, or simply a consequence of secondary carbohydrate metabolism.

#### 2.2.2. Molecular Mechanisms: Gene Expression and Biosynthetic Control in Fruits

Although fructan biosynthesis has been extensively characterized in vegetative storage organs, its molecular regulation in fleshy fruits remains poorly understood. The core biosynthetic pathway is highly conserved across fructan-producing species and relies on members of the glycoside hydrolase family 32 (GH32), primarily *sucrose:sucrose fructosyltransferase* (*1-SST*), *fructan:fructan fructosyltransferase* (*1-FFT*), and *sucrose:fructan 6-fructosyltransferase* (*6-SFT*), together with fructan exohydrolases (FEHs) responsible for fructan degradation. While these genes are broadly conserved among monocots and dicots, fructan accumulation depends not simply on their presence but on the coordinated temporal and spatial regulation of their expression, enzyme activity, and substrate availability [4,26,37].

Current evidence suggests that the absence of substantial fructan accumulation in most fleshy fruits results primarily from regulatory constraints rather than from the loss of biosynthetic genes. In banana, transient induction of *1-SST* during ripening coincides with the accumulation of short-chain FOS, whereas limited or absent *1-FFT* expression restricts further polymer elongation [38,39]. Likewise, only weak or transient expression of fructosyltransferase genes has been reported in strawberry and tomato, where fructan concentrations remain close to analytical detection limits [40,41]. By contrast, coordinated expression of *1-SST*, *1-FFT*, and *6-SFT* characterizes classical fructan-accumulating organs such as chicory roots, asparagus spears, onion bulbs, and agave reproductive tissues, resulting in sustained fructan biosynthesis and accumulation [26,35,37,43].

Regulation of fructan metabolism integrates hormonal, metabolic, and developmental signals. Promoter analyses have identified ABA-, auxin-, ethylene-, and sugar-responsive cis-elements in fructosyltransferase genes, indicating that fructan biosynthesis is tightly coupled to developmental programs and environmental cues [26]. Sucrose functions not only as the substrate for fructan synthesis but also as a signaling molecule capable of activating *1-SST* through hexokinase- and *SnRK1*-dependent pathways, whereas *trehalose-6-phosphate* and *TOR* signaling appear to repress fructan biosynthesis under conditions favoring rapid carbon utilization [37,43]. In cereals, additional transcriptional regulation is mediated by *AP2*/*ERF* and *bZIP* transcription factors that coordinate sugar and stress responses with fructan accumulation [44]. Collectively, these findings support the hypothesis that fructan biosynthesis is governed by an integrated regulatory network rather than by individual genes acting independently.

A major unresolved question is why this conserved regulatory machinery is rarely activated in fleshy fruits. One possibility is that fruit development prioritizes sucrose and hexose metabolism over long-term carbon storage, thereby limiting fructan biosynthesis despite the presence of conserved GH32 genes. Another possibility is that fructan metabolism occurs only in specific cell types or developmental stages, remaining below the detection limits of conventional analytical approaches. Future studies combining tissue-resolved transcriptomics, metabolomics, enzyme localization, and functional genomics will be essential to determine whether fructan biosynthesis in fruits represents a tightly regulated adaptive mechanism, an evolutionary remnant, or a latent metabolic pathway that can be activated under specific physiological or environmental conditions.

### 2.3. Evolutionary Perspectives: Why Do Certain Lineages Accumulate Fructans in Reproductive Organs?

The phylogenetic distribution of fructan metabolism across angiosperms is markedly discontinuous, reflecting repeated, independent evolutionary events rather than inheritance from a single fructan-accumulating ancestor. Both the presence of fructans in reproductive structures (fruits, grains, inflorescences) and their absence in most fleshy fruits are outcomes of lineage-specific metabolic evolution shaped by ecological pressures, life-history strategies, and genomic plasticity.

#### 2.3.1. Phylogenetic Distribution and Convergent Evolution

Fructan metabolism occurs in approximately 15% of angiosperm families, prominently within *Asteraceae*, *Poaceae*, *Asparagaceae*, *Campanulaceae*, and *Boraginaceae* [22,37]. This patchy distribution implies multiple independent origins of fructan biosynthesis, a pattern further supported by biochemical and phylogenomic evidence (Figure 1D).

Early phylogenetic analyses identified fructan-active enzymes as derivatives of vacuolar and cell wall invertases, originating through gene duplication and neofunctionalization within the glycoside hydrolase family 32 (GH32) [4,37]. Subsequent comparative studies demonstrated that distinct lineages have recruited separate subsets of GH32 genes for fructan synthesis, leading to the evolution of inulin-type pathways in dicots (*Asteraceae*, *Campanulaceae*) and graminan/levan-type pathways in monocots (*Poaceae*, *Asparagaceae*) [1,45].

This evolutionary mosaic is mirrored in fruits and reproductive structures. Low but consistent fructan or FOS accumulation has been reported in fruits of *Musa* spp., *Fragaria* × *ananassa*, *Malus domestica*, and *Pyrus communis* [38,39,42], species that phylogenetically fall within families where fructan metabolism is largely absent or vestigial. Such findings suggest that ancestral GH32 enzymes may have retained low-level fructosyltransferase activity, enabling the synthesis of short-chain FOS without full fructan biosynthetic competence.

Comparative genomics has further revealed that fructan-related GH32 genes persist as latent orthologs in many fruit-producing species. For example, *Solanum lycopersicum* and *Malus domestica* retain *1-SST*-like and *1-FFT*-like genes exhibiting conserved catalytic residues but minimal expression in fruit tissues [41]. This supports a model in which the capacity for fructan synthesis was ancestrally widespread, but tissue-specific regulatory repression during the evolution of fleshy fruits led to its near disappearance.

Recent genomic analyses provide direct evidence of convergent enzyme evolution. In *Asteraceae*, *Cichorium intybus* fructosyltransferases independently acquired fructan-active motifs via convergent amino acid substitutions [27], while in monocots, *Agave* species evolved unique β (2 → 6)-levan-type enzymes distinct from *Poaceae 6-SFTs* [34,35]. These independent trajectories illustrate how fructan metabolism has repeatedly arisen as an adaptive response to environmental stress and carbon storage requirements, with reproductive fructan allocation emerging secondarily in some lineages.

#### 2.3.2. Organ-Specific Carbon Allocation and Evolutionary Strategies

The distribution of fructans among plant organs reflects fundamental evolutionary trade-offs in carbon allocation, resource storage, and reproductive investment rather than random species-specific variation. Across fructan-producing plants, two broad allocation strategies can be recognized. Perennial species, including *Cichorium intybus*, *Helianthus tuberosus*, and *Smallanthus sonchifolius*, preferentially accumulate inulin-type fructans in underground storage organs such as roots and tubers, where they support overwintering, vegetative regrowth, and long-term buffering against environmental stress [4,26]. In these taxa, fructans function as stable carbon reserves that enhance plant persistence, whereas reproductive organs generally contain negligible amounts of these polymers.

**Figure 2:**
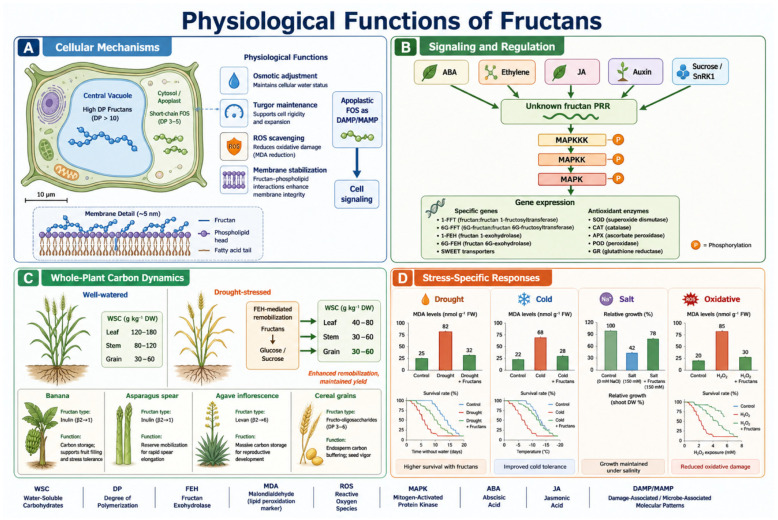
Multifaceted physiological roles of fructans in plant stress adaptation, signaling, and carbon allocation. (**A**) Cellular-level functions of fructans and fructooligosaccharides (FOS). High-degree polymerization (DP) fructans accumulate predominantly in the vacuole as reserve carbohydrates, whereas short-chain FOS may occur in the apoplast and cytosol. These molecules contribute to osmotic adjustment, maintenance of cell turgor, reactive oxygen species (ROS) scavenging, and membrane stabilization through interactions with phospholipid bilayers. Apoplastic FOSs have been proposed to function as signaling molecules acting as DAMP/MAMP-like compounds; however, their perception mechanisms and downstream signaling pathways remain hypothetical and require direct experimental validation. (**B**) Conceptual signaling model summarizing putative regulatory networks associated with fructan metabolism. Environmental and hormonal signals, including abscisic acid (ABA), ethylene, jasmonic acid (JA), auxin, and sucrose/SnRK1 pathways, may activate signaling cascades involving MAP kinases and transcriptional regulation of fructan biosynthesis- and degradation-related genes, as well as antioxidant defense systems. The proposed roles of fructan perception, MAPK activation, and hormone crosstalk are inferred from currently available evidence and should be considered working hypotheses rather than experimentally established mechanisms. (**C**) Role of fructans in whole-plant carbon dynamics. Under favorable conditions, fructans accumulate in vegetative tissues as water-soluble carbohydrate reserves, whereas under drought stress they are remobilized through fructan exohydrolase (FEH) activity to sustain metabolism, grain filling, and yield stability. Representative examples of fructan accumulation and function in banana, asparagus, agave, and cereal crops are shown. (**D**) Schematic summary of fructan-mediated responses to major abiotic stresses, including drought, cold, salinity, and oxidative stress. Fructan accumulation is associated with reduced membrane damage, improved survival, enhanced stress tolerance, and maintenance of growth under adverse environmental conditions. Where indicated, proposed mechanisms are based on current evidence but remain to be fully validated, particularly those involving signaling functions and thermotolerance. The figure integrates evidence synthesized from the literature reviewed in this study.

In contrast, annual cereals such as wheat, barley, and rye exhibit a markedly different allocation strategy. Rather than maintaining long-term carbohydrate reserves, fructans accumulate transiently in stems and developing grains, where they serve as remobilizable carbon pools that sustain grain filling when photosynthetic activity declines under drought or other environmental constraints [33,36] (Figure 2C). This reproductive allocation strategy reflects strong evolutionary selection for maximizing seed production under fluctuating environmental conditions and illustrates how fructan metabolism has been integrated into distinct life-history strategies.

Intermediate patterns also occur in species where fructan metabolism has been partially recruited into reproductive tissues. In *Musa* spp., transient expression of 1-SST and 1-FFT during early fruit ripening is accompanied by the temporary accumulation of short-chain FOS [36,37], suggesting that remnants of the fructan biosynthetic pathway may have been retained and co-opted for osmotic regulation during ripening rather than long-term carbohydrate storage. Likewise, *Agave tequilana* and *A. americana* accumulate levan-type fructans in inflorescences, where these carbohydrates appear to support flowering and reproductive development under arid environmental conditions [34]. Collectively, these examples indicate that fructan metabolism in reproductive organs is not the result of independent evolutionary innovations but rather of lineage-specific redeployment of an ancestral vegetative pathway under different developmental and hormonal regulatory programs.

This evolutionary framework also provides a plausible explanation for the limited occurrence of fructans in fleshy fruits. Unlike storage organs or cereal grains, fleshy fruits have evolved primarily to promote seed dispersal through the rapid accumulation of soluble sugars that enhance sweetness, attract dispersal agents, and sustain the high metabolic demands associated with ripening. Under this scenario, fructan biosynthesis appears to have been progressively reduced or transcriptionally repressed in most fruit lineages, whereas sucrose, glucose, and fructose became the dominant forms of carbohydrate storage. Whether this represents a complete evolutionary loss or a latent pathway that remains inducible under specific developmental or environmental conditions remains an important unresolved question.

## 3. Physiological Functions of Fructans and FOS: Cellular Mechanisms, Metabolic Integration, and Stress Adaptation

Fructans and fructooligosaccharides (FOS) are increasingly recognized as multifunctional carbohydrates whose biological significance extends well beyond their traditional role as reserve compounds. Although they share a common biosynthetic origin, their physiological functions differ markedly according to their degree of polymerization (DP), molecular architecture, and tissue localization. High-DP fructans primarily contribute to long-term carbon storage, osmotic adjustment, membrane stabilization, and protection against environmental stresses, whereas short-chain FOSs are more readily mobilized and are emerging as signaling molecules involved in stress perception, defense priming, and metabolic communication. This functional specialization provides a conceptual framework for understanding how structurally related carbohydrates participate in distinct but complementary aspects of plant adaptation.

Accumulating evidence indicates that fructan metabolism integrates cellular homeostasis with environmental responsiveness. Beyond acting as reserve carbohydrates, fructans participate in osmotic regulation, redox buffering, membrane protection, and carbon remobilization, while FOSs increasingly appear to function as regulators of stress signaling and physiological plasticity (Figure 2D). Together, these complementary roles position fructan metabolism as a dynamic component of plant resilience, linking metabolic flexibility with developmental and environmental adaptation.

### 3.1. Established Physiological Functions of Long-Chain Fructans

#### 3.1.1. Osmotic Regulation and Carbon Buffering

The best-established physiological function of fructans is their contribution to osmotic adjustment under conditions that restrict cellular water availability. Because fructans accumulate predominantly within the vacuole, they behave as compatible solutes that increase osmotic potential without interfering with essential biochemical reactions. Their remarkable solubility and hydration capacity enable plants to maintain cellular turgor during dehydration caused by drought, salinity, or freezing [6,18].

Besides their osmoprotective role, fructans constitute highly dynamic carbon reserves that buffer fluctuations between carbon supply and demand. This function is particularly evident in temperate cereals, where stems accumulate large quantities of water-soluble carbohydrates, of which fructans may account for nearly 80% during grain filling [46,47]. During post-anthesis drought or heat stress, when photosynthetic activity declines, these reserves are rapidly remobilized to sustain grain development and reproductive success [31,48]. Consequently, fructans operate not merely as passive storage polymers but as flexible metabolic reservoirs that stabilize carbon allocation under fluctuating environmental conditions.

This buffering capacity depends on the coordinated activity of fructan-synthesizing enzymes (1-SST, 1-FFT, and 6-SFT) and fructan exohydrolases (FEHs), which together regulate reversible carbon fluxes between sucrose and fructan pools. Rather than representing static carbohydrate deposits, fructan reserves continuously adjust according to developmental stage, source-sink relationships, and environmental constraints. The substantial variation observed among tissues, developmental stages, and genotypes therefore reflects an adaptive strategy that optimizes carbon economy under stress [47,49].

#### 3.1.2. Membrane and Protein Stabilization

In addition to their osmotic functions, fructans directly contribute to preserving cellular integrity during dehydration and low-temperature stress. Experimental evidence indicates that fructan polymers interact with phospholipid membranes through hydrogen bonding with polar head groups, generating hydrated molecular networks that reduce membrane phase transitions during water loss [50,51]. These interactions help preserve membrane fluidity, decrease lipid peroxidation, and maintain enzyme functionality under freezing or severe dehydration.

Interestingly, these protective properties appear to depend strongly on polymer architecture. Tamura et al. (2014) [52] demonstrated that transgenic *Brachypodium distachyon* plants expressing the timothy grass *PpFT1* (*6-SFT*) gene accumulated higher-DP fructans and exhibited significantly greater freezing tolerance than plants expressing the wheat *TaFT2* gene, which predominantly produces shorter fructans. These findings support the hypothesis that longer fructan polymers promote the formation of vitrified amorphous matrices capable of stabilizing membranes and proteins during freeze-induced dehydration.

Additional evidence comes from cold-acclimated wheat, where Chowhan et al. (2024) [53] reported that fructans and starch are enzymatically converted into soluble carbohydrates during freezing stress. Besides contributing to osmotic adjustment, these soluble sugars reduce freezing point depression and reinforce membrane stability. Winter cultivars capable of accumulating greater amounts of fructans and soluble sugars consistently display enhanced freezing tolerance, highlighting the dual function of fructans as both structural protectants and readily mobilizable carbon reserves.

Despite these compelling physiological observations, the precise molecular basis of fructan-membrane interactions remains incompletely understood. Most current models derive from indirect physiological evidence or in vitro experiments, whereas direct visualization of fructan–lipid interactions inside living cells is still lacking. Future studies employing high-resolution approaches—including solid-state NMR spectroscopy, differential scanning calorimetry, cryo-electron microscopy, and molecular dynamics simulations—will be necessary to validate these proposed stabilization mechanisms and determine how fructan chain length influences membrane behavior under stress.

#### 3.1.3. Antioxidant Activity and Redox Homeostasis

In addition to the osmotic protection, fructans also contribute to maintaining cellular redox homeostasis. Although they are not considered classical antioxidant molecules, numerous studies demonstrate that fructans possess significant hydroxyl radical-scavenging activity, thereby reducing oxidative damage associated with drought, salinity, cold, and other abiotic stresses [2,54].

The antioxidant properties of fructans are closely linked to their molecular structure. Terminal fructose residues provide reactive sites capable of neutralizing reactive oxygen species (ROS), while increased chain length and branching appear to enhance radical-scavenging efficiency [24]. Consequently, high-DP fructans generally display greater antioxidant potential than shorter fructooligosaccharides, reinforcing the concept that polymer architecture determines biological function.

An additional layer of complexity arises from fructan turnover during stress. The activation of fructan exohydrolases (FEHs) simultaneously releases soluble fructose molecules and short-chain FOS that may function as compatible solutes or signaling compounds. Thus, fructan degradation represents not simply carbon mobilization but a coordinated metabolic transition linking energy supply, osmotic adjustment, antioxidant protection, and stress signaling. This remarkable versatility illustrates how fructan metabolism integrates multiple physiological processes into a unified adaptive response rather than functioning solely as carbohydrate storage.

### 3.2. Emerging Mechanisms: Fructans and FOS as Signaling and Regulatory Molecules

Although the protective functions of fructans as reserve carbohydrates are well established, increasing evidence indicates that their biological activity extends beyond osmotic buffering and carbon storage. Importantly, not all fructan molecules behave similarly. High-degree polymerization (DP) fructans primarily contribute to structural protection and long-term carbon sequestration, whereas short-chain fructooligosaccharides (FOS) and fructan-derived oligosaccharides appear to function as signaling molecules capable of modulating stress responses. This distinction suggests that polymer size is not only a structural characteristic but also a determinant of biological function, providing an additional layer of metabolic regulation.

One of the most compelling emerging concepts is the role of fructans and FOS in stress priming. Rather than directly activating defense pathways, several fructan-derived oligosaccharides prepare plant tissues for a faster and stronger response to subsequent environmental challenges. Levan oligosaccharides (LOS), particularly sulfated forms, induce rapid apoplastic reactive oxygen species (ROS) production characteristic of elicitor-triggered responses, whereas non-sulfated LOS enhance resistance by priming antioxidant and defense pathways without causing major metabolic disruption [55,56]. These observations support the “Sweet Immunity” concept, whereby soluble carbohydrates participate in immune signaling in addition to their metabolic roles.

Current evidence further suggests that FOS may function as damage-associated molecular patterns (DAMPs) generated during stress-induced fructan degradation. In this model, fructan hydrolysis by fructan exohydrolases (FEHs) not only releases carbon substrates but also produces signaling oligosaccharides capable of coordinating local and systemic defense responses. Because FEH activity increases during dehydration, cold stress, senescence, and tissue remodeling, fructan catabolism may simultaneously provide metabolic substrates and activate adaptive signaling pathways, integrating carbon remobilization with stress perception.

Despite these advances, the molecular basis of fructan perception remains largely unresolved. Two non-exclusive models have been proposed. The first suggests direct recognition by plasma membrane pattern-recognition receptors (PRRs), analogous to receptors involved in the perception of chitin, β-glucans, or oligogalacturonides. The second proposes indirect sensing through alterations in osmotic status, cellular redox balance, or energy metabolism, which subsequently activate ABA-, JA-, or MAPK-dependent signaling cascades [26,47]. Experimental evidence supporting the latter includes the observation that nystose induces ABA-responsive genes and MAPK signaling during cold acclimation in rice and Arabidopsis, suggesting that specific FOS molecules may function as mobile metabolic signals linking carbohydrate status with transcriptional reprogramming [47]. Nevertheless, no bona fide fructan receptor has yet been identified, representing one of the most important unresolved questions in fructan biology.

Hormonal signaling further contributes to the integration of fructan-mediated responses. Rather than controlling fructan biosynthesis—which is discussed in Section 2.2.2—phytohormones appear to modulate the physiological consequences of fructan turnover. ABA coordinates osmotic adaptation during drought, ethylene promotes fructan degradation during senescence and fruit ripening, and jasmonates regulate fructan remodeling under wounding and biotic stress [26,57] (Figure 2B). These interactions position fructans at the interface between carbohydrate metabolism and hormonal signaling networks, allowing plants to integrate developmental cues with environmental information.

An emerging concept is that fructans should be viewed as dual-function metabolites, whose biological role depends largely on their polymer size and metabolic context. Long-chain fructans mainly provide carbon storage, membrane stabilization, and osmoprotection, whereas short-chain FOS generated either during biosynthesis or controlled degradation appear to act as signaling molecules involved in stress priming, immune activation, and metabolic communication. Understanding how plants regulate the transition between these structural and signaling pools represents a major challenge for future research and will likely determine how fructan metabolism can be exploited to improve crop resilience under climate change.

## 4. Fructans and FOS: Implications for Human Nutrition and Health

Fructans and FOS have emerged as multifunctional dietary fibers with broad physiological relevance. Their capacity to modulate gut microbiota composition, immune function, and host metabolism positions them at the interface of nutrition, microbiology, and health science. Extensive evidence indicates that these prebiotic carbohydrates promote the proliferation of beneficial bacteria—particularly *Bifidobacterium* and *Lactobacillus*—while stimulating the production of short-chain fatty acids (SCFAs), key metabolites linking microbial activity to systemic metabolic and immune outcomes [10,58,59]. The biological activity of fructans is strongly determined by their molecular structure, especially the degree of polymerization (DP), which governs fermentation kinetics, microbial selectivity, and physiological tolerability [60,61].

### 4.1. Mechanisms of Action: A Unified Dual-Pathway Framework

Fructans and FOS exert their effects through two complementary pathways: (i) microbiota-dependent fermentation, which mediates most metabolic and gastrointestinal benefits, and (ii) microbiota-independent interactions, involving direct modulation of host immune and epithelial cells. This duality provides a unifying conceptual framework that connects prebiotic functionality with systemic health outcomes.

#### 4.1.1. Microbiota-Dependent Pathway

The primary mechanism involves selective fermentation by gut bacteria, notably *Bifidobacterium* and *Lactobacillus* [10,58]. The process follows a sequential cascade:

FOS intake → Selective fermentation → SCFA production → Systemic physiological effects.

SCFAs—acetate, propionate, and butyrate—mediate downstream signaling by binding to G-protein coupled receptors (GPR41, GPR43, and GPR109A) on intestinal and immune cells, regulating glucose metabolism, lipid homeostasis, and inflammation [62,63]. Butyrate also enhances intestinal barrier integrity by serving as the main energy source for colonocytes and by tightening epithelial junctions [64]. These mechanisms explain the bifidogenic and anti-inflammatory properties of fructans, as well as their contributions to metabolic regulation and gut homeostasis [59,65].

#### 4.1.2. Microbiota-Independent Pathway

Parallel evidence demonstrates that fructans can directly interact with host immune cells. Certain *β*-(2,1)-linked fructans modulate Toll-like receptors (TLR2, TLR4), influencing NF-κB signaling and cytokine production in macrophages and dendritic cells [66]. They can also engage pattern-recognition receptors, such as C-type lectins, and stimulate intestinal epithelial cells to secrete mucins and antimicrobial peptides [58,67]. These direct effects may explain the rapid immune modulation observed shortly after fructan ingestion, preceding major microbiota shifts [68]. The extent to which each pathway contributes depends on fructan structure, dosage, and host immune status (Table 2).

### 4.2. Structure–Function Relationships: The Central Role of Degree of Polymerization (Dp)

#### 4.2.1. Dp-Dependent Functional Spectrum

The DP is the principal structural determinant of fructan bioactivity. Chain length defines fermentation rate, selectivity, and host responses across microbial, immune, metabolic, and technological dimensions (Table 3).

Medium-chain FOSs (DP 6–15) represent a “functional optimum,” balancing rapid fermentation with sustained bifidogenicity [10,61]. In contrast, long-chain inulins (DP > 15) exert superior immunomodulatory and metabolic effects, likely due to their ability to reach distal colon segments, sustain butyrate production, and interact with immune receptors [63,71].

#### 4.2.2. The Microbial Diversity Paradox

Interestingly, short-term supplementation with short-chain FOS (DP 2–10) can transiently reduce overall microbial diversity despite promoting beneficial taxa such as *Bifidobacterium* [58], whereas longer-chain inulins tend to enhance or stabilize diversity [60]. This pattern reflects differential substrate selectivity and temporal dynamics of microbial adaptation. Reduced diversity under short-term exposure does not necessarily imply functional loss, as key metabolic capacities are often maintained through redundancy [72]. These findings emphasize the need to prioritize functional rather than purely compositional microbiome metrics in assessing prebiotic efficacy.

### 4.3. Inter-Individual Variability and Personalized Nutrition

A striking feature of the fructan and FOS literature is the substantial inter-individual variability in physiological responses, ranging from profound metabolic improvements and microbiota shifts in some individuals to minimal or negligible effects in others [58,72]. This heterogeneity, often dismissed as experimental noise, represents a critical biological phenomenon with profound implications for precision nutrition strategies.

#### 4.3.1. Determinants of Response Variability

Human responses to fructan intake vary widely due to microbial, genetic, and dietary factors [58,72]. Baseline microbiota composition is the most consistent predictor: individuals with low *Bifidobacterium* abundance generally exhibit stronger bifidogenic and metabolic responses, while those with high baseline levels show attenuated effects [58]. Metagenomic studies reveal that the abundance of *β*-fructofuranosidase and fructose transporter genes correlates with fermentation efficiency and SCFA yield [72].

Host genetic factors further modulate responsiveness—polymorphisms in TLR2/TLR4 and SCFA receptor genes (*GPR41*, *GPR43*) can alter immune and metabolic sensitivity to fructan-derived signals [66,71]. Age and physiological state also influence outcomes: infants show heightened bifidogenic responses due to microbiota composition dominated by *Bifidobacterium* [73], whereas older adults often require higher doses or longer interventions due to reduced microbial diversity [64].

#### 4.3.2. Toward Predictive and Personalized Strategies

Integrating these determinants enables the identification of biomarkers predictive of fructan responsiveness. Table 4 summarizes key candidates spanning microbiota composition, genetic polymorphisms, metabolic phenotype, and dietary context. Such predictive frameworks can guide individualized dietary interventions and inform biotechnological design of fructan-enriched crops optimized for population-specific needs.

Dietary context remains a key modulator—low-fiber diets enhance prebiotic efficacy, while high-fiber backgrounds can diminish additional effects [74]. These determinants collectively underscore the importance of personalized nutrition approaches that consider microbiome, host, and diet as interdependent variables shaping fructan efficacy.

### 4.4. Translational Biotechnology and Integrated Nutritional Design

#### 4.4.1. Designer Fructan Crops

The intersection of plant physiology and human nutrition creates a unique biotechnological opportunity: developing crops with fructan profiles optimized for both stress tolerance and health promotion. Fructans act as osmoprotectants and membrane stabilizers in plants while functioning as prebiotics and metabolic modulators in humans [6,21,26]. Optimizing their DP distribution bridges these dual functions—medium-chain fructans (DP 6–15) enhance osmotic adjustment and selective bifidogenicity, while long-chain inulins (DP > 20) improve membrane stability, glycemic control, and immune modulation [63,71].

Achieving this integration requires metabolic engineering of key enzymes (1-SST, 1-FFT, 6G-FFT, 1-FEH) to fine-tune polymer length, alongside breeding and post-harvest optimization strategies that preserve structural integrity [23,64]. This approach establishes fructans as molecular bridges between plant adaptation and human well-being—an archetype for “dual-function” metabolic engineering.

#### 4.4.2. Integrated Nutritional Design Framework

Fructans exemplify how integrated nutritional design can unify agronomic resilience and human nutrition through shared metabolic pathways. By modulating fructan structure—especially DP—crops can be tailored to thrive under abiotic stress while enhancing human metabolic and digestive health [2,6]. As summarized in Table 5, targeted DP profiles allow for simultaneous optimization of plant function and health benefits.

## 5. Fructan and FOS Biotechnology: Genetic Engineering and Crop Improvement

Fructans and FOS represent a strategic nexus between plant stress physiology, human nutrition, and biotechnology. Their biosynthesis involves the sequential transfer of fructosyl units from sucrose to acceptor molecules, catalyzed by sucrose:sucrose 1-fructosyltransferase (1-SST), fructan:fructan 1-fructosyltransferase (1-FFT), and sucrose:fructan 6-fructosyltransferase (6-SFT). The coordinated action of these enzymes—counterbalanced by fructan exohydrolases (1-FEH)—determines both the total fructan pool and its degree of polymerization (DP).

This vacuolar pathway is dynamically regulated by developmental and environmental cues such as drought, temperature, and photoperiod, linking fructan metabolism to both plant stress adaptation and nutritional functionality [32,75].

### 5.1. Genetic Engineering Strategies

Modern biotechnology has expanded the ability to modulate fructan biosynthesis with precision, allowing simultaneous improvement of plant performance and functional compound yield. Table 6 provides an overview of representative strategies, target systems, and outcomes, outlining how different genetic approaches influence fructan accumulation, structural diversity, and stress-related phenotypes while identifying key challenges that remain for large-scale implementation.

Overexpression of biosynthetic genes such as *Ta1SST* and *Ta6SFT* enhances fructan accumulation—particularly high-DP forms—under drought and osmotic stress, conferring improved resilience and carbohydrate storage stability [32,76].

Conversely, suppression of degradative enzymes like *1-FEH* reduces fructan turnover and prolongs post-harvest stability [77].

Gene stacking approaches combining *1-SST*, *1-FFT*, and *6G-FFT* provide fine control of DP distributions, enabling crops with specific structural profiles optimized for both osmoprotection and human health outcomes [26,27].

Pathway reconstruction in non-fructan species such as rice and maize has demonstrated successful accumulation of inulin-type fructans and enhanced chilling tolerance [44,78].

At the microbial level, metabolic engineering of FOS-producing strains (e.g., deletion of *sacC* in *Zymomonas mobilis*) has yielded up to nine-fold increases in levan production through optimized substrate channeling [79,80].

### 5.2. Genetic Engineering Versus Conventional Breeding

Quantitative trait loci and QTNs linked to fructan metabolism have been identified in cereals and forage crops, enabling marker-assisted selection of favorable alleles controlling DP distribution and total fructan content [81,82].

The comparative analysis of fructan improvement approaches reveals distinct yet complementary strengths across breeding, transgenesis, and genome editing. Conventional breeding remains indispensable for capturing polygenic variation, environmental stability, and local adaptation, although progress is inherently slow and constrained by existing allelic diversity. Transgenic engineering, in contrast, enables the introduction of novel or heterologous genes such as *1-SST*, *1-FFT*, and *6-SFT* to dramatically enhance fructan biosynthesis and tailor polymer length, but it faces regulatory, pleiotropic, and consumer acceptance challenges (Table 6). Genome editing technologies, particularly CRISPR/Cas9, bridge these approaches by offering precise, transgene-free modifications of endogenous genes, allowing fine-tuning of expression and metabolic flux within native regulatory contexts. Collectively, these strategies are not mutually exclusive but synergistic—integrating genomic selection, GWAS-informed allele discovery, and targeted editing provides a powerful framework for designing crops with optimized fructan content, controlled degree of polymerization, and enhanced multi-stress resilience [6,26,27].

### 5.3. Alternative Production Systems

In recent years, microbial biofactories have emerged as powerful and versatile platforms for fructan and FOS production, complementing and, in some cases, surpassing plant-based systems in precision, yield, and scalability [49,77,79,83]. Engineered microorganisms such as *Zymomonas mobilis*, *Aspergillus niger*, and *Lactobacillus reuteri* exemplify successful applications of synthetic biology and metabolic engineering to achieve high-yield, structure-specific fructan synthesis. For example, targeted deletion of the *sacC* gene in *Z. mobilis* eliminated competing invertase activity, leading to a ninefold increase in levan accumulation (≈45 g/L) with high molecular weight (DP 50–200), suitable for functional food and pharmaceutical applications [79,80]. Similarly, recombinant *A. niger* strains expressing heterologous fructosyltransferases or inulosucrases have achieved industrial-level production of short-chain FOS (DP 3–10) exceeding 100 g/L, representing one of the most successful microbial routes for prebiotic ingredient manufacturing [82]. In parallel, *L. reuteri* and related lactic acid bacteria have been utilized for in situ FOS synthesis during the fermentation of dairy and cereal matrices, generating synbiotic foods that integrate probiotic activity with intrinsic prebiotic enrichment [58,84].

These microbial systems provide key advantages over plant-based production, including tighter control over DP, improved product purity, and continuous operation independent of environmental constraints [58,79,83]. Although initial capital and energy inputs are higher, microbial fermentation eliminates the need for agricultural land, supports modular scaling, and enables the synthesis of tailor-made FOS with defined physicochemical and biological properties [80,83]. Conversely, plant systems—such as chicory, Jerusalem artichoke, and cereals—remain superior for large-scale, low-cost inulin production and carbon-negative value chains [6,21,26]. Integrating both strategies offers a promising hybrid model: plants serving as feedstocks or enzyme sources, and microbes as precision biocatalytic systems for structural refinement [77,84].

### 5.4. Fruits as Model Systems for Integrated Fructan Improvement

As shown in Table 7, selected fruit species differ in their natural fructan content, DP profiles, stress susceptibility, and genetic engineering feasibility, providing complementary model systems for integrated fructan improvement. Collectively, these examples illustrate a continuum of opportunity—from crops with existing fructan metabolism suitable for enhancement (banana, yacón) to those requiring pathway introduction (apple, tomato). The choice of model thus depends on the specific research objective: validating biosynthetic control mechanisms, improving stress resilience, or developing consumer-facing functional varieties.

Advancing this field requires answering several key research questions. First, it is essential to determine which genes and regulatory elements govern natural variation in FOS accumulation among cultivars [26,33]. Second, experimental evaluation must clarify whether fruit-specific overexpression of *1-SST* or *1-FFT* affects sensory attributes such as sweetness or texture, potentially necessitating compensatory breeding for sugar composition [40]. Third, the hypothesis that fructans can extend post-harvest shelf life through osmoprotective and antioxidant functions should be systematically tested in controlled storage studies [85]. Finally, the metabolic trade-offs of enhanced fructan biosynthesis—such as competition with soluble sugars, vitamins, or secondary metabolites—must be assessed through metabolomic profiling and multi-environment trials [6].

### 5.5. Trade-Offs, Pleiotropic Effects, and Agronomic Considerations

Despite the considerable progress in engineering fructan metabolism, the successful agricultural deployment of modified genotypes hinges on a nuanced understanding of physiological trade-offs, pleiotropic effects, and agronomic stability. Elevated fructan or FOS accumulation inevitably competes with other carbon sinks, and its modulation can influence yield, quality, and environmental adaptability. This subsection critically examines these challenges and outlines key research priorities to ensure translational success.

#### 5.5.1. Carbon Allocation Trade-Offs

Increasing fructan synthesis demands significant carbon investment, often diverting sucrose from essential pathways supporting growth, respiration, and secondary metabolism. Empirical studies reveal that while enhanced fructan content can improve abiotic stress tolerance, it may also reduce biomass or yield in certain contexts. For instance, overexpression of *6-SFT* in tall fescue resulted in a 15–20% biomass reduction linked to constrained carbon flow toward cell wall formation [32], while co-expression of *1-SST* and *1-FFT* in potato led to diminished starch accumulation and lower tuber yield [29]. Conversely, some *Cichorium intybus* lines overexpressing *1-SST* exhibited minimal yield penalty, highlighting the importance of genetic background in modulating these effects [77].

Experimental quantification of these trade-offs should include multi-environment yield trials and ^13^C-based carbon partitioning analyses to assess how fructan accumulation impacts resource allocation under both optimal and stress conditions. Conditional expression systems, where fructan biosynthesis is induced by drought or cold, could help balance protective benefits with yield preservation [26].

#### 5.5.2. Palatability and Sensory Quality

Fructan biosynthesis consumes free sugars, potentially reducing sweetness—a critical trait in consumer-facing crops. Evidence from tuber and fruit systems suggests that high fructan levels can lower soluble sugar content, altering texture and flavor [29]. These sensory trade-offs are particularly relevant in fruit crops targeted for direct consumption (Section 5.4). To mitigate them, strategies include selecting high-sugar genetic backgrounds, modulating fructan DP to influence perceived sweetness, and leveraging consumer education around “low-glycemic” or “prebiotic” benefits.

Systematic sensory evaluation and metabolomic profiling of engineered fruits, including *Musa* and *Fragaria*, will be crucial to determine thresholds of fructan enrichment compatible with consumer acceptability [40,85].

#### 5.5.3. Pleiotropic Effects and Metabolic Coupling

Beyond primary carbon trade-offs, genetic perturbation of fructan pathways can trigger pleiotropic effects through shared metabolic intermediates or regulatory crosstalk. Overexpression of transcription factors such as *CiMYB17* in chicory, for example, simultaneously activated biosynthetic and degradative enzymes, complicating efforts to achieve stable fructan accumulation [90]. Similarly, *1-SST* overexpression in heterologous systems altered amino acid profiles, reflecting broader impacts on nitrogen–carbon metabolism [26].

Multi-omics approaches integrating transcriptomic, metabolomic, and phenomic data are essential to detect such unintended consequences. Comparative profiling across developmental stages and environments can reveal metabolic imbalances or compensatory mechanisms influencing performance stability [6,21].

#### 5.5.4. Stability Across Environments

Fructan accumulation and transgene expression frequently vary with environmental conditions due to promoter sensitivity, substrate availability, and epigenetic regulation. Field studies in perennial ryegrass showed 2–5-fold variation in fructan levels among sites [75], and transgene silencing has been observed in 10–20% of *Cichorium* lines after several generations [77]. The use of native promoters, single-copy insertions, and targeted genomic integration sites can enhance expression stability. Multi-location, multi-year trials remain indispensable for evaluating genotype × environment (G × E) interactions before commercial release [32].

#### 5.5.5. Agronomic and Ecological Implications

At the ecosystem level, modified fructan metabolism may alter rhizosphere interactions, pest dynamics, and input requirements. Increased root fructan can influence microbial communities, potentially enhance beneficial symbioses or alter nutrient cycling. High-fructan plants may also exhibit different palatability to herbivores, affecting pest resistance [91]. Agronomically, modified carbon partitioning could shift nitrogen demand, water-use efficiency, and optimal harvest timing.

To address these dimensions, integrative field experiments are required—combining microbiome sequencing, insect bioassays, and factorial agronomic trials (fertilization × irrigation × harvest stage)—to define management practices that sustain both productivity and environmental compatibility [92].

### 5.6. Regulatory Barriers and Public Perception

The successful deployment of fructan-engineered crops increasingly depends on regulatory acceptance and societal perception rather than technical feasibility. Regulatory frameworks for genetically modified organisms (GMOs) and genome-edited plants differ widely across jurisdictions, shaping both innovation pace and market potential [93,94].

#### 5.6.1. Regulatory Landscape

Two main paradigms dominate: process-based systems, typified by the European Union, regulate any organism modified by recombinant or editing technologies, resulting in prolonged approval timelines and mandatory labeling [3,95]. In contrast, product-based systems—used in the United States, Canada, Brazil, and Argentina—evaluate the trait rather than the method, often exempting genome-edited plants lacking foreign DNA from GMO regulation [96].

This divergence creates a dual opportunity: while transgenic fructan crops face long regulatory delays, CRISPR-mediated edits (e.g., *FEH* knockout, *1-SST* modulation) can advance rapidly under simplified oversight, enabling faster commercialization and broader acceptance [97].

#### 5.6.2. Public Acceptance

Consumer attitudes toward engineered crops remain strongly influenced by perceived benefit and naturalness. Acceptance rises substantially when modifications deliver direct health advantages—such as improved digestive or metabolic health—rather than agronomic traits [98,99]. Fructan-enriched fruits and cereals fit this category, allowing communication framed around nutritional enhancement of natural compounds rather than artificial modification.

Genome editing technologies also benefit from greater trust, often perceived as “precision breeding” rather than transgenesis [100]. Effective strategies include transparent communication, institutional partnerships, and positive labeling emphasizing prebiotic function rather than breeding method.

#### 5.6.3. Emerging Opportunity: Genome Editing and NGTs

New genomic techniques (NGTs), such as base and prime editing, enable site-specific nucleotide changes without foreign DNA insertion [101]. These methods already qualify as non-GMO in several regions—including the United States, Japan, Brazil, and Argentina—and may soon gain similar status in the European Union. For fructan biotechnology, they represent a regulatory and societal inflection point, combining molecular precision with consumer-aligned “natural” narratives.

## 6. Future Perspectives

Domestication has profoundly reshaped plant carbohydrate metabolism by favoring traits associated with yield, palatability, storage performance, and postharvest quality. Consequently, fructan metabolism has been modified differently across crop groups according to their agronomic value and selection history. In cereals such as Triticum and Hordeum, breeding has primarily targeted grain yield and starch accumulation, leading to reduced fructan concentrations in mature grains despite the persistence of considerable natural variation within wild relatives and landraces [23]. Conversely, crops such as chicory and Jerusalem artichoke have been specifically selected for enhanced fructan storage capacity and increased polymerization degree, making them the principal commercial sources of inulin [27].

Fruit crops appear to have followed a distinct evolutionary and breeding trajectory. Selection in species such as *Musa*, *Malus*, and *Fragaria* has consistently favored higher concentrations of soluble sugars to improve sweetness and consumer acceptance. This process likely reduced the selective advantage of maintaining active fructan biosynthesis during fruit development, contributing to the weak expression of fructosyltransferase genes observed in modern cultivars [40,41]. Importantly, however, the persistence of GH32 homologs in these genomes suggests that the genetic potential for fructan biosynthesis has not been eliminated but instead may have become transcriptionally restricted during domestication.

This observation opens new perspectives for crop improvement. Rather than introducing entirely novel metabolic pathways, modern breeding and genome engineering could exploit endogenous fructan biosynthetic genes to increase FOS accumulation in fruits while simultaneously improving tolerance to abiotic stress and postharvest deterioration. Promoter editing of native *GH32* genes could enable fruit-specific activation of fructan biosynthesis without disrupting carbohydrate partitioning in vegetative tissues. Likewise, targeted modification of *FEH* genes may reduce fructan degradation and increase net polymer accumulation, whereas allele introgression from fructan-rich wild relatives could broaden the enzymatic diversity available for breeding programs.

Beyond improving nutritional quality through increased prebiotic content, these strategies may also enhance osmotic regulation, oxidative stress tolerance, and fruit shelf life, thereby linking plant physiology with human nutrition. Consequently, fructan metabolism represents an attractive target for next-generation biofortification programs aimed not only at increasing functional food value but also at developing climate-resilient fruit crops. Achieving this objective will require integrating comparative genomics, functional genetics, metabolomics, and precision breeding to better understand how carbon allocation pathways can be reprogrammed without compromising fruit quality or consumer acceptance.

## 7. Conclusions

Fructans and fructooligosaccharides (FOS) represent a versatile group of plant-derived carbohydrates that connect plant metabolism, stress physiology, and human nutrition. Their structural diversity and dynamic metabolism contribute to plant adaptation to adverse environmental conditions while simultaneously providing compounds with recognized prebiotic and health-promoting properties. Despite substantial progress in understanding fructan metabolism in vegetative tissues, their occurrence and functional roles in fruits remain comparatively understudied. Exploring fructan biosynthesis in fruit tissues may open new opportunities for improving fruit nutritional quality and developing novel functional foods. Recent advances in genomics, metabolic engineering, and synthetic biology provide powerful tools to manipulate fructan biosynthesis and tailor carbohydrate profiles in crops. Future research integrating plant physiology, biotechnology, and nutritional science will be essential to fully exploit the ecological and biotechnological potential of fructans and FOS. Such interdisciplinary approaches may ultimately contribute to the development of climate-resilient crops and innovative food products with enhanced health benefits.

## Figures and Tables

**Table 1:** Structure–Function Matrix of Plant Fructans.

Fructan Type	Dominant Linkage Pattern	Typical Lineages (Plant Families)	Characteristic DP Range	Primary Physiological Roles	Adaptive/Environmental Association	Representative References
Inulin-type	β (2 → 1) linear	Dicotyledons—*Asteraceae*, *Campanulaceae*, *Boraginaceae*	3–60	Osmotic adjustment, ROS scavenging, membrane stabilization	Drought and cold tolerance in temperate species	[1,2,17,22,24,26,30,34,35]
Levan-type	β (2 → 6) branched	Monocotyledons—*Agavaceae*, *Asparagaceae*, *Poaceae* (desert/thermophilic species)	3–200	Hydration shell stability, enzyme and membrane protection, osmotic buffering	Heat and desiccation tolerance in arid ecosystems	
Graminan/Mixed-type	β (2 → 1) + β (2 → 6) mixed (branched)	Temperate grasses—*Poaceae* (*Triticum*, *Hordeum*, *Secale*)	3–40	Dual osmotic and structural buffering, carbon remobilization during grain filling	Multi-stress adaptation (fluctuating drought/temperature)	[23,32,33,36]
Neo-inulin/Neo-levan	β (2 → 1) or β (2 → 6) with glucose branch at O6	Diverse (e.g., *Allium*, *Asparagus*, *Agave*)	3–30	Transitional storage and metabolic flexibility	Developmental and stress-linked regulation	[26,37]

**Table 2:** Mechanistic Pathways of Fructan and FOS Action.

Mechanism	Observed Effect	DP Requirement	Human Evidence	Animal Evidence	Knowledge Gaps
MICROBIOTA-DEPENDENT PATHWAY					
Bifidogenic effect	↑ *Bifidobacterium* abundance (2–3 log CFU/g)	DP 2–10 optimal; DP > 10 moderate	Strong (multiple RCTs, *n* = 20–60) [10,58]	Very strong (consistent across models)	Dose–response curves; ceiling effects; inter-individual variability predictors
SCFA production	↑ Acetate, propionate, butyrate (20–40% increase in fecal concentrations)	DP 3–20; longer chains → more butyrate	Moderate (variable correlations with metabolic outcomes) [59,65]	Strong (consistent dose-dependent increases)	Portal vs. systemic SCFA levels; tissue-specific effects; temporal dynamics
Microbial diversity modulation	Variable: ↑ diversity (long-chain), ↓ diversity (short-term FOS)	DP >15 generally promotes diversity	Mixed (depends on baseline microbiota, duration) [58,60]	Mixed (model-dependent)	Functional redundancy vs. diversity; resilience metrics; long-term stability
Gut barrier function	↓ Intestinal permeability; ↑ mucin production	DP 5–60	Moderate (limited direct human studies) [64]	Strong (multiple IBD models) [69]	Mechanisms beyond SCFA; mucin glycosylation patterns
MICROBIOTA-INDEPENDENT PATHWAY					
TLR modulation	↓ NF-κB activation; altered cytokine profiles	DP 10–30 more potent	Limited (few human studies) [66]	Moderate (in vitro + in vivo) [67]	Structure–activity relationships; receptor specificity; in vivo relevance
Dendritic cell maturation	Altered DC phenotype; ↑ regulatory DC populations	DP > 10	Very limited (small human trials)	Moderate (murine models) [68]	Dose–response; tissue compartment specificity; clinical significance
IgA secretion enhancement	↑ Mucosal and systemic IgA (15–30% increase)	DP 10–60	Moderate (several human trials, variable results) [58,70]	Strong (consistent across models)	Microbiota-dependent vs. independent contributions; antigen specificity
Epithelial signaling	↑ Antimicrobial peptides; ↑ mucin gene expression	DP 5–30	Limited (in vitro human cell lines)	Moderate (ex vivo tissue models)	Signaling pathways; receptor identification; physiological concentrations

Note: ↑ and ↓ indicate increases and decreases, respectively, in metabolite accumulation, enzyme activity, gene expression, physiological traits, or other evaluated parameters relative to the corresponding control or reference condition.

**Table 3:** Degree polymerization (DP)-dependent functional specialization of fructans and fructooligosaccharides: current evidence, mechanistic basis, and research gaps.

Functional Domain	Short-Chain FOS (DP 2–5)	Medium-Chain Fructans (DP 6–15)	Long-Chain Fructans (DP > 15)	Current Level of Evidence	Knowledge Gaps
Microbiota modulation	Rapid fermentation by *Bifidobacterium*; proximal colon	Balanced fermentation and cross-feeding	Slow fermentation reaching distal colon; broader microbial diversity	Strong	Standardized human studies comparing identical doses across DP classes
SCFA production	Mainly acetate	Balanced acetate-propionate	Greater butyrate production	Strong	Relationship between DP and microbiome composition remains incompletely resolved
Immune regulation	Mostly indirect through SCFA	Moderate immunomodulation	Stronger IgA induction and TLR modulation	Moderate	Direct molecular receptors remain unidentified
Metabolic health	Acute glycemic effects	Intermediate metabolic benefits	Sustained lipid lowering and insulin sensitivity	Moderate	Long-term randomized clinical trials
Digestive tolerance	Greater gas production and bloating	Intermediate tolerance	Better tolerated despite higher doses	Strong	Influence of microbiome adaptation over time
Food technology	High sweetness and solubility	Intermediate functionality	Fat replacement, gel formation and thermal stability	Strong	Optimization according to food matrix
Plant physiological relevance	Signaling molecules; stress priming	Mixed structural and signaling roles	Osmoprotection, membrane stabilization and carbon storage	Moderate	Precise DP thresholds separating signaling from structural functions

The information presented represents an integrated synthesis based on the literature, particularly [2,6,18,24,31,47,48,50,52,54,55,56,57].

**Table 4:** Candidate Biomarkers for Predicting Fructan Response.

Biomarker Category	Specific Markers	Predicted Outcome	Evidence Level	Implementation Feasibility
Microbiota Composition	Baseline *Bifidobacterium* abundance	Inverse correlation with bifidogenic response (ceiling effect)	Strong (multiple studies)	High (16S rRNA sequencing; qPCR)
	Baseline *Faecalibacterium* abundance	Positive correlation with butyrate production	Moderate	High
	Enterotype (*Prevotella* vs. *Bacteroides*)	Differential SCFA profiles	Moderate	Moderate (requires deeper sequencing)
	Microbiota diversity (Shannon index)	Variable; low diversity may predict larger compositional shifts	Mixed evidence	High
Microbiota Functional Capacity	β-fructofuranosidase gene abundance	Fermentation efficiency; SCFA production capacity	Moderate (limited studies)	Moderate (metagenomic sequencing)
	Fructose transporter genes	Substrate uptake efficiency	Limited	Moderate
	Butyrate synthesis pathway genes	Butyrate production potential	Moderate	Moderate
Host Genetics	*GPR41*/*GPR43* SNPs	SCFA receptor sensitivity; metabolic responses	Limited (candidate gene studies)	Moderate (genotyping)
	*TLR2*/*TLR4*/*NOD2* SNPs	Immune response magnitude	Limited	Moderate
	*FTO*, *PPAR-γ* variants	Metabolic outcome variability	Speculative	Moderate
Metabolic Phenotype	Baseline insulin sensitivity (HOMA-IR)	Greater improvement in insulin-resistant individuals	Moderate	High (clinical chemistry)
	Fasting glucose, HbA1c	Glycemic response prediction	Moderate	High
	Inflammatory markers (CRP, IL-6)	Anti-inflammatory effect magnitude	Moderate	High
	BMI, waist circumference	Weight management response	Moderate	Very high
Dietary Assessment	Habitual fiber intake	Inverse correlation with response magnitude	Moderate	High (dietary assessment tools)
	Fermented food consumption	May predict enhanced fermentation capacity	Limited	High
Immune Markers	Baseline IgA levels	Inverse correlation with IgA enhancement	Limited	Moderate (ELISA)
	Regulatory T cell frequency	Immune modulation potential	Limited	Low (flow cytometry)

**Table 5:** Integrated design objectives linking plant function and human health through fructan modulation.

Plant Function	Human Health Benefit	Target Fructan Profile	Expected Outcomes
Enhanced drought tolerance	Improved glycemic control	↑ Medium-chain FOS (DP 8–15) ↑ Long-chain inulin (DP 15–30)	Reduced irrigation requirements Lower post-prandial glucose Enhanced satiety
Osmotic stress protection	Bifidogenic prebiotic effect	↑ Total fructan content (5–10% DW) Optimized DP 6–15 range	Improved survival under water stress Maximal Bifidobacterium promotion Digestive health benefits
Cold tolerance	Metabolic syndrome management	↑ Long-chain inulin (DP > 20)	Extended growing season/geography Enhanced lipid metabolism effects Weight management support

Note: ↑ indicates increased fructan or fructooligosaccharide (FOS) accumulation.

**Table 6:** Genetic engineering strategies for fructan and FOS biosynthesis in plants and microorganisms.

Strategy	Target Genes/Enzymes	Organisms/Crops	Outcome	Limitations
Gene overexpression	*Ta1SST*, *Ta6SFT*	Wheat, barley	↑ High-DP fructan; ↑ drought tolerance	Variable field stability
FEH suppression	*1-FEH*	Chicory, barley	↓ Fructan degradation; ↑ post-harvest stability	Carbon turnover alterations
Gene stacking	*1-SST* + *1-FFT* + *6G-FFT*	Wheat, ryegrass	Tailored DP profiles; ↑ stress resilience	Regulatory complexity
Synthetic pathway reconstruction	Wheat genes → rice/maize	Rice, maize	Novel fructan accumulation; ↑ chilling tolerance	Metabolic burden
Microbial engineering	*sacC* deletion	*Z. mobilis*	↑ Levan yield (×9)	Industrial scale-up constraints

Note: ↑ and ↓ indicate increases and decreases of the fructan or fructooligosaccharide (FOS) accumulation.

**Table 7:** Candidate fruit crops for fructan engineering and functional improvement.

Crop	Native Fructan Status	Stress Context	Engineering Feasibility	Strategic Research Focus	References
Banana (*Musa* spp.)	Moderate FOS (2–5% DW, DP 3–8)	Drought, post-harvest ripening	Transformation established; genome sequenced	Overexpress 1-SST/1-FFT; suppress FEH; evaluate fructan stability during ripening and stress tolerance	[85,86]
Strawberry (*Fragaria* × *ananassa*)	Low FOS (<1% DW, DP 3–5)	Water stress, oxidative decay	Routine transformation; *F. vesca* as model	Engineer fructan accumulation to improve shelf life and firmness	[40]
Apple (*Malus domestica*)	Trace levels (<0.1% DW)	Drought, storage disorders	Transformation feasible; grafting allows propagation	Introduce fructan biosynthetic pathway; test storage quality and disorder reduction	[87]
Tomato (*Solanum lycopersicum*)	Absent	Salinity, post-harvest softening	Well-established transformation system	Introduce 1-SST/1-FFT; evaluate effects on sweetness, texture, and stress tolerance	[88]
Yacón (*Smallanthus sonchifolius*)	High FOS (10–15% FW, DP 3–20)	Frost, root rot	Limited transformation tools; high natural variation	Characterize germplasm; improve agronomic performance through selection	[89]

## Data Availability

No new data were created or analyzed in this study.
